# Characterization of saltiness-enhancing peptides from *Pleurotus eryngii*: identification, sensory evaluations, and mechanism of saltiness-enhancing

**DOI:** 10.1038/s41538-025-00681-8

**Published:** 2026-02-07

**Authors:** Min Yang, Wei Wang, Biyang Zhu, Changli Zeng, Aimin Ma, Hongbo Wang, Danyun Xu

**Affiliations:** 1 College of Life Science, Jianghan University, Wuhan, Hubei China; 2 College of Light Industry and Food Engineering, Guangxi University, Nanning, China; 3 College of Food Science and Technology, Huazhong Agricultural University, Wuhan, China

**Keywords:** Biochemistry, Biotechnology, Computational biology and bioinformatics, Drug discovery

## Abstract

This study aimed to identify novel saltiness-enhancing peptides derived from *Pleurotus eryngii* and evaluate their influence on saltiness perception. Utilizing an integrated virtual screening strategy, 6 candidate peptides exhibiting potential saltiness-enhancing properties were identified. Sensory analysis revealed that these peptides displayed distinct taste profiles, with detection thresholds ranging between 0.04 and 0.12 mmol/L. Notably, peptides AGHDDFP, GYDTF, and NGYDMR enhanced the saltiness of a 3 mg/mL NaCl solution, demonstrating synergistic or additive effects, consistent with electronic tongue. Molecular docking analysis revealed that three saltiness-enhancing peptides primarily interacted with TMC4 through hydrogen bonding, identifying key interaction residues including Gln527, Glu531, Asp491, Asn404, Arg437, Lys567, Pro409, and Val498. Subsequent molecular dynamics simulations confirmed the structural stability and tightness of saltiness-enhancing peptides-TMC4 complexes, supporting their potential effectiveness in modulating saltiness perception. These results indicate a promising approach for identifying saltiness-enhancing peptides derived from *Pleurotus eryngii*, potentially serving as taste modulators in reduced-sodium food formulations.

## Introduction

Saltiness, a fundamental taste, plays an integral role in daily diets. Predominantly, table salt, primarily composed of sodium chloride (NaCl), served as the chief source of saltiness in foods. When consumed, NaCl dissociated into sodium and chloride ions, which contributed to essential physiological functions such as osmotic pressure maintenance and metabolic regulation^1^. Current global NaCl consumption averaged between 9 and 12 g daily, significantly exceeding the World Health Organization’s (WHO) recommendation of no more than 6 g^2^. This excessive intake of NaCl was linked to severe health issues, including cardiovascular diseases, hypertension, and end-organ damage^3^. Consequently, the food industry has prioritized reducing NaCl content in foods to foster healthier eating habits. This reduction often diminished the food’s palatability and introduced taste anomalies^4^. Since taste was a pivotal factor in consumer preference and acceptance^5^, the industry now prioritized strategies that lower sodium levels without compromising saltiness or quality. To achieve this, various strategies have been employed, such as optimizing the shape and size of salt crystals, utilizing salt substitutes, and incorporating taste enhancers^6^. Nonetheless, these methods could lead to suboptimal taste experiences, textural issues, potential health hazards for older adults or those with compromised potassium excretion, and increased production costs, limiting their practicality for broad market adoption^7^. An emerging and promising approach involved the exploration of saltiness and saltiness-enhancing peptides. These peptides are natural, nutritious, and readily absorbed by the body, potentially changing the way NaCl intake is managed in the food industry. Enzymatic hydrolysis is the predominant approach for generating active peptides. Animal-derived proteins, particularly from meat, eggs, dairy, and seafood, remain the main raw material. However, rising demand for animal protein, together with environmental, ethical, and health concerns linked to livestock production^8^, highlights the need for high quality alternative feedstocks to enable scalable development and commercialization of taste active peptides.

*Pleurotus eryngii* is a mushroom species commercially cultivated and extensively distributed throughout China and other Asian nations. Renowned for its delectable taste, distinct aroma, and rich nutrient content, including proteins and peptides, this mushroom served as a good raw material for developing flavorful products^9^. Given the prominent umami taste of edible mushrooms, the majority of existing research has concentrated on the exploration of umami peptides in various species, such as *Agaricus bisporus, Boletus edulis, and Stropharia rugoso-annulata*. However, investigations into peptides that impart or enhance saltiness are notably scarce. To date, there have been no studies on the extraction of saltiness-enhancing peptides from *P. eryngii*. The identification of saltiness-enhancing peptides derived from *P. eryngii* lays a foundation for the development of novel, naturally sourced food additives. This progress may facilitate the formulation of a range of saltiness-enhancing products derived from natural resources. Accordingly, an in-depth investigation into the saltiness-enhancing properties of *P. eryngii* could explore the peptide composition, their interaction mechanisms with taste receptors, and further our understanding of the diversity of saltiness-enhancing agents present in mushrooms.

Traditionally, the identification of taste-active peptides has involved sequential steps of isolation, purification, structural characterization, and eventual chemical synthesis to confirm their taste properties. These conventional techniques typically require extensive time and labor, thereby impeding the rapid identification of novel taste-active peptides^10^. Research on peptides capable of enhancing saltiness perception remains in preliminary stages, and current insights into the structure-activity relationships of these peptides are still limited. Consequently, these limitations significantly hinder efficient and accurate identification of target peptides within large-scale peptide databases derived from protein hydrolysates. Recent findings have demonstrated that umami-active compounds could enhance saltiness perception. Additionally, research into the structure-activity relationships of umami peptides has advanced considerably in recent years^7^. Multiple machine learning methodologies, such as Umami-SCM, UMPred-FRL, Umami_YYDS, and Umami-MRNN, have been effectively utilized for umami peptide predictions^11^. In the present investigation, integrated predictive models were applied to preliminarily screen novel umami peptides that show promise as saltiness-enhancing agents. Subsequent verification processes facilitated the swift and efficient discovery of novel salt-enhancing peptides. Conducting sensory evaluations of these saltiness-enhancing peptides is essential to ascertain their taste impact in both aqueous solutions and food matrices. Given the dynamic characteristics in taste perception, the implementation of dynamic sensory evaluation methodologies has become essential to accurately characterize consumer perceptions during consumption. For instance, the time-intensity (TI) method provided quantitative information regarding peak taste intensity, the timing of this peak, its duration, and the continuous variations in sensory perception occurring over the evaluation period^12^. Furthermore, the application of electronic tongue technology, capable of providing objective and reproducible evaluations, has emerged as valuable tools in taste research^13^.

In 2021, transmembrane channel-like protein 4 (TMC4) was identified as a chloride channel implicated in oral saltiness perception, although alternative mechanisms remain under investigation^14^. In mice, loss of TMC4 markedly reduced glossopharyngeal responses to high concentration NaCl. This attenuation, characterized by decreased neural firing and impaired signal transmission, supported a mechanistic role for TMC4 in saltiness transduction. Accordingly, recent peptide studies (e.g., from yeast proteins and *Lentinula edodes*) have used TMC4 as the functional receptor to probe saltiness enhancement^7,15,16^. On this basis, we selected TMC4 as the receptor for the present study.

The study of ligand-receptor interactions, which were important in activating taste receptors, has become complex due to the intricate nature of food matrices. Molecular docking and molecular dynamics simulations (MDS) have proven effective in addressing this complexity. Molecular docking enabled the determination of optimal binding modes and sites through energetically favorable interactions^17^. Molecular docking alone was insufficient to fully capture the conformational changes occurring in both receptors and ligands during their interactions. In comparison, MDS effectively addressed this limitation and has been widely utilized to explore the interaction mechanisms and conformational dynamics of receptor-ligand complexes, thereby enhancing our comprehension of these sophisticated molecular events^18^.

In this study, we extracted peptide fractions from *P. eryngii* through enzymatic hydrolysis. Subsequently, we analyzed the peptide with molecular weights below 3k Da utilizing mass spectrometry. Peptides with potential to enhance saltiness were identified through virtual screening techniques. The properties of these saltiness-enhancing peptides in enhancing saltiness and their interactions with TMC4 were examined through sensory evaluation, electronic tongue, molecular docking, and MDS. This study both broadens the repertoire of saltiness-enhancing peptides available for additive applications and deepens the investigation of taste-active constituents in enzymatic hydrolysis products of *P. eryngii*.

## Results and discussion

### Taste characterization of *P. eryngii* hydrolysate (<3k Da) (PEU)

Figure S1 indicated that PEU primarily presented saltiness and umami, accompanied by slight sweetness and sourness, achieving umami intensity rating of 3.50. Previous research reported that low-molecular-weight components (<3 kDa) derived from *L. edodes* possessed significant taste intensity^16^. Considering its marked umami, PEU was deemed appropriate for the identification of potential saltiness-enhancing peptides and subsequent experimental analyses.

### Virtual screening of potential saltiness-enhancing peptides in PEU

Previous studies have established the efficacy of umami peptides in augmenting saltiness perception^7,19,20^. Therefore, this research systematically screened potential umami peptides derived from PEU to identify those possessing saltiness-enhancing capabilities. In total, 5984 peptides were characterized from PEU. Many studies have emphasized that shorter peptides, typically comprising fewer than ten amino acids, showed significant taste-enhancing effects^21^. Additionally, PeptideRanker values, indicating the likelihood of peptide bioactivity, were utilized; scores above 0.5 signified substantial bioactive potential^10^. In the current investigation, 1822 peptides shorter than ten amino acids and PeptideRanker scores exceeding 0.5 were selected for further evaluation. Ensuring the safety of peptides and minimizing the risk of toxicity or allergenic reactions in food industry applications necessitates early-stage evaluations. This preventive strategy is essential for identifying and excluding peptides with potential adverse effects, thereby protecting consumer health. The computational tools ToxinPred and AllerTOP, recognized widely for their dependability, were utilized in this research to systematically assess the toxicity and allergenicity of 1822 peptides. The analysis revealed that 954 peptides were predicted as non-toxic, among which 692 were also classified as non-allergenic. Additionally, characteristics such as water solubility and peptide stability are important determinants of their efficacy and practical applicability. According to predictions by the Innovagen tool, 506 peptides exhibited favorable water solubility. Moreover, the Expasy-ProtParam tool forecasted that 302 peptides possess adequate stability for potential food industry usage^22^. These peptides were further validated for umami characteristics utilizing machine learning models, including Umami-SCM, UMPred-FRL, Umami_YYDS, and Umami-MRNN. Employing multiple predictive algorithms enhanced reliability and accuracy, compensating for individual model constraints, thereby improving the robustness of umami peptide identification^23,24^. Concordance across these diverse models increased confidence in the identified peptides’ potential umami characteristics. Predictions were further verified through the BIO-UWM sensory module for peptides and amino acids. Ultimately, following this comprehensive screening protocol, only 6 peptides (AGHDDFP, GYDTF, NGYDMR, FSDY, EMMETPPGF, and SPTP) from the initial set of 5984 were identified as promising saltiness-enhancing candidates for subsequent synthesis and experimental validation. The MS/MS spectra corresponding to these selected peptides were presented in Fig. S2.

### Taste characteristics of synthetic peptides

Table 1 demonstrated that all 6 identified peptides exhibiting potential saltiness-enhancing properties concurrently displayed umami attributes, each presenting different taste profiles. This complexity in taste perception could be attributed to the peptides’ amino acid composition and chemical characteristics^7^. Specific structural regions within these peptides selectively interact with various taste receptors, thereby creating different sensory response^25^. Aspartic acid (D) and glutamic acid (E) were recognized as a major contributor to umami, whereas amino acids associated with sweetness, such as alanine (A) and glycine (G), also played significant roles. In contrast, hydrophobic amino acids, including leucine (L) and phenylalanine (F), were correlated with bitter sensations^26^. The sensory detection thresholds of the 6 peptides in aqueous solutions varied distinctly. AGHDDFP exhibited the lowest threshold at 0.04 mmol/L, followed by EMMETPPGF at 0.06 mmol/L. The peptides NGYDMR and SPTP both presented thresholds at 0.08 mmol/L, while GYDTF had a threshold of 0.10 mmol/L, and FSDY exhibited the highest value at 0.12 mmol/L. These threshold concentrations were comparable to those previously reported for saltiness-enhancing peptides derived from *Stropharia rugosoannulat*a, ranging from 0.11 to 0.55 mmol/L^22^.

**Table 1:** Taste characteristics and thresholds of peptides

Peptides	taste characteristic	taste threshold (mmol/L)
AGHDDFP	Umami, sourness, saltiness, slight bitterness	0.04
GYDTF	Umami, sourness	0.10
NGYDMR	Umami, sourness, sweetness	0.08
FSDY	Umami, sourness	0.12
EMMETPPGF	Umami, sweetness, astringency	0.06
SPTP	Umami, sourness, bitterness, astringency	0.08

### QDA analysis

**Fig. 1:**
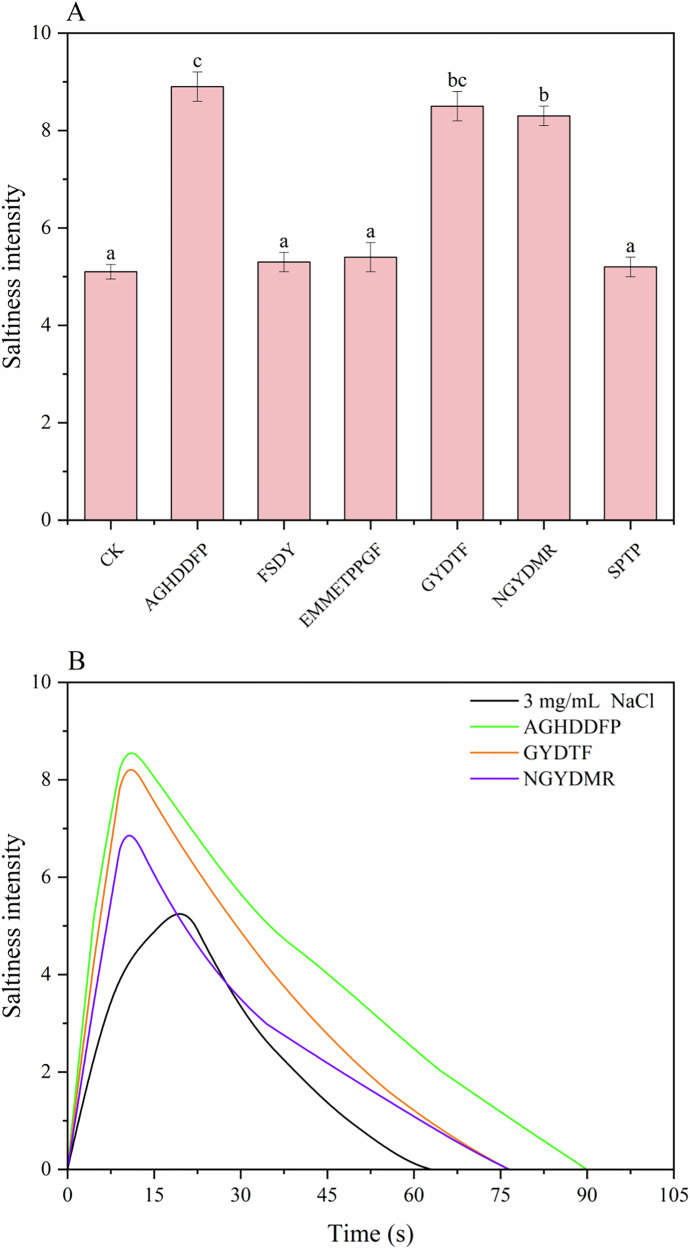
Saltiness-enhancing effects of 6 peptides on 3 mg/mL NaCl. **A** Quantitative descriptive analysis of 6 peptides on 3 mg/mL NaCl; **B** the time−intensity curve of 3 saltiness enhancing peptides on 3 mg/mL NaCl.

The saltiness-enhancing effects of 6 peptides (1 mg/mL) were assessed by incorporating them into a 3 mg/mL NaCl solution. Figure 1A demonstrated that peptides AGHDDFP, GYDTF, and NGYDMR, at a concentration of 1 mg/mL, significantly enhanced the saltiness intensity of the NaCl solution, whereas the impacts of the other peptides were less distinct. Notably, AGHDDFP most substantially increased the saltiness intensity, with GYDTF and NGYDMR following. This enhancement could be primarily ascribed to interactions involving saltiness-enhancing peptides and NaCl. Similarly, Niu et al.^7^ reported that supplementing a 3 mg/mL NaCl solution with 1 mg/mL of six peptides (NKF, LGLR, WDL, NMKF, FDSL, and FDGK) significantly increased perceived saltiness intensity. Due to their significant effects in enhancing saltiness, AGHDDFP, GYDTF, and NGYDMR were selected for further investigation to determine their enhancement characteristics and underlying mechanisms.

### Time-intensity (TI)analysis

Figure 1B presented the temporal profiles of saltiness intensity for the control sample (3 mg/mL NaCl, CK) and the three saltiness-enhancing peptides. While the general pattern of the curves remained consistent across all samples, those with saltiness-enhancing peptides exhibited a rapid ascent to peak intensity followed by a gradual decline, effectively enhancing and sustaining the sensation of saltiness within the oral cavity. Table S2 displayed pronounced disparities in the I _max_ among the samples: CK (5.50), AGHDDFP (8.83), GYDTF (8.58), and NGYDMR (7.25), demonstrating significant enhancement of saltiness intensity by the saltiness-enhancing peptides. Additionally, the *T*_ext_ varied substantially: CK showed 75 s, compared to 102, 90, and 90 s for AGHDDFP, GYDTF, and NGYDMR, respectively, indicating the saltiness-enhancing peptides’ effectiveness in extending the saltiness duration. Meanwhile, there were differences in the time to reach *T*_max_, with CK at 30 s versus 20 s for the saltiness-enhancing peptides. This reduction suggested that the saltiness-enhancing peptides accelerated the perception of peak saltiness intensity, attributable to a marked increase in rate of intensity rise (*R*_increase_). The AUC varied significantly, with CK showing a lower AUC of 504 compared to the considerably higher values of 1195, 892, and 698 for AGHDDFP, GYDTF, and NGYDMR, respectively. This discrepancy in AUCs could be attributed to the saltiness-enhancing peptides’ rapid amplification of saltiness intensity, resulting in larger overall AUC. The results presented in Fig. 4A and Table S2 indicated that supplementation with the three saltiness-enhancing peptides (1 mg/L) significantly enhanced both the perceived intensity and duration of saltiness compared to the control over the entire assessing period. Schindler et al.^27^ have previously reported that arginyl dipeptides could significantly enhance the saltiness intensity of NaCl solutions ranging from 2.3 to 3.8 mg/mL, consistent with the findings of this study.

### Interactions of saltiness-enhancing with NaCl

**Fig. 2:**
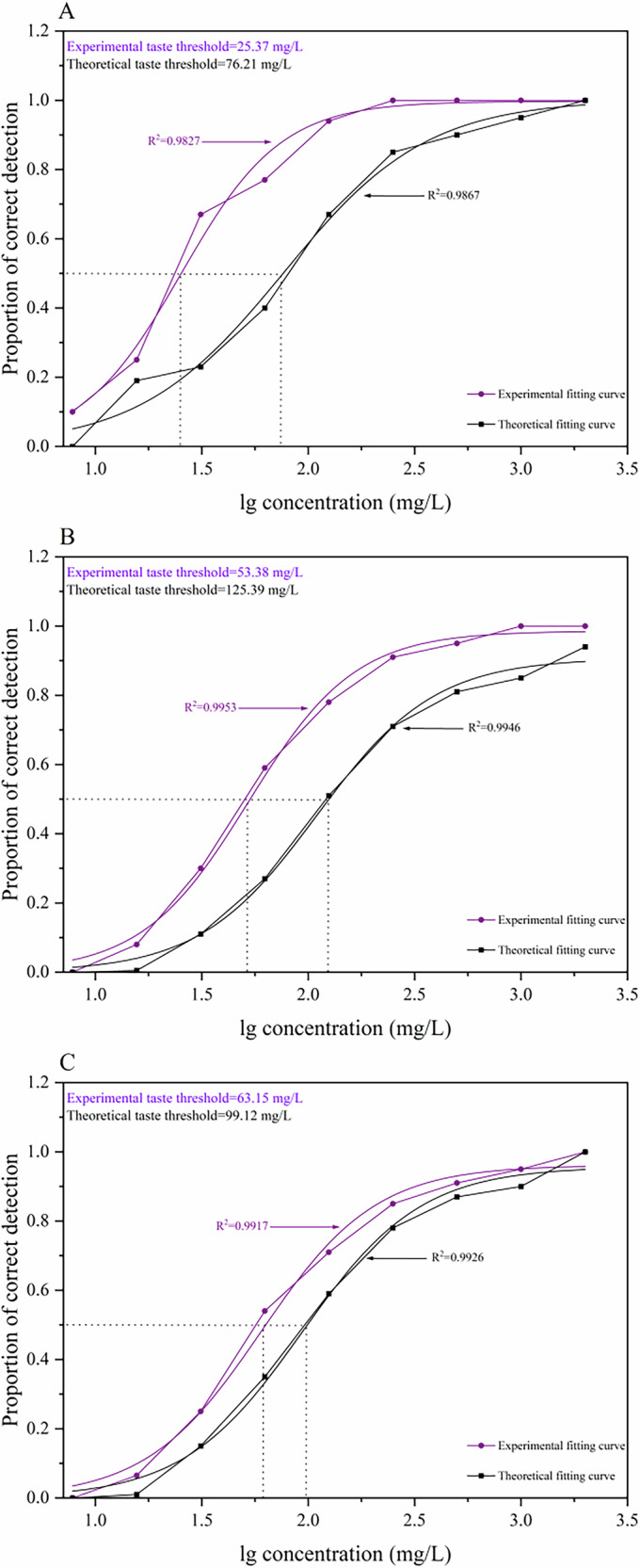
Saltiness enhancement results of peptide and NaCl interaction models. **A** Detection thresholds change of NaCl mixed with AGHDDFP. **B** Detection thresholds change of NaCl mixed with GYDTF. **C** Detection thresholds change of NaCl mixed with NGYDMR.

The interactions among AGHDDFP, GYDTF, NGYDMR, and NaCl were investigated utilizing triangle testing combined with sigmoidal curve fitting, facilitating the determination of both theoretical and experimental detection thresholds. The ratio of experimental to theoretical detection thresholds was defined as the *D*-value. A *D*-value exceeding 1 indicated a masking effect exerted by the saltiness-enhancing peptides, while a *D*-value equal to 1 implied an absence of interaction. Values between 0.5 and 1 indicated additive interactions, whereas values equal to or below 0.5 reflected synergistic interactions^19^. As illustrated in Fig. 2, the *D*-values obtained for AGHDDFP, GYDTF, and NGYDMR with NaCl were 0.33, 0.43, and 0.64, respectively. These findings demonstrated that the inclusion of these peptides enhanced saltiness perception through synergistic interactions (AGHDDFP and GYDTF) as well as additive interaction (NGYDMR). Consequently, these saltiness-enhancing peptides exhibit potential as taste modulators for sodium reduction strategies, enabling lower salt content in food products while retaining desirable sensory characteristics.

### Electronic tongue analysis

**Fig. 3:**
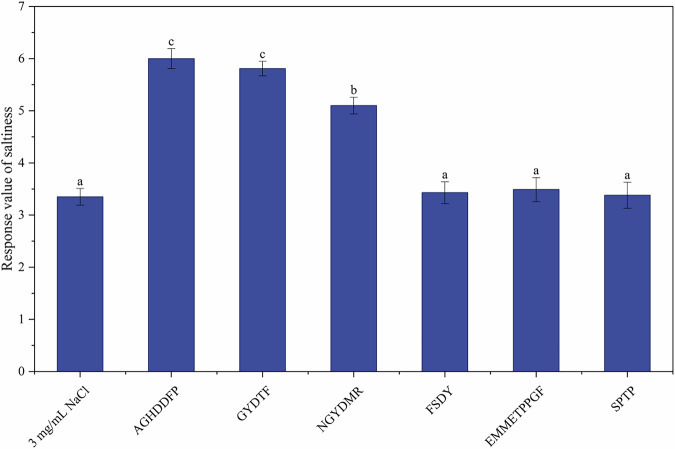
Response value of saltiness based on electronic tongue of 3 saltiness-enhancing peptides on 3 mg/mL NaCl. Electronic tongue results of six peptides.

This study used an electronic tongue as an electrochemical proxy to assess concordance between instrument outputs and sensory evaluations. By transducing taste stimuli into electrical signals, the system provides objective taste profiling and permits quantitative comparison with sensory evaluation. For the 3 mg/mL NaCl control, the mean response was 3.6. With AGHDDFP, GYDTF, and NGYDMR, responses rose to 6.4, 5.3, and 5.6, respectively, whereas the remaining peptides did not differ significantly from the control (Fig. 3). The electronic tongue results were consistent with the sensory results. Similarly, Cao et al.^28^ reported that VESQTNGIIR, NQITKPNDVY, and DEDTQAMP significantly enhanced the perceived saltiness of NaCl solutions using electronic tongue.

### Molecular docking

We conducted molecular docking analyses on three peptides (AGHDDFP, GYDTF, and NGYDMR), previously identified through sensory evaluation as significantly enhancing saltiness. This study involved constructed TMC4 homology modeling, subsequently validated via Ramachandran plots, as depicted in Fig. S3, with 100% of the residues found within permissible regions. This confirmed the reliability of the homology models used in subsequent docking analyses.

**Fig. 4:**
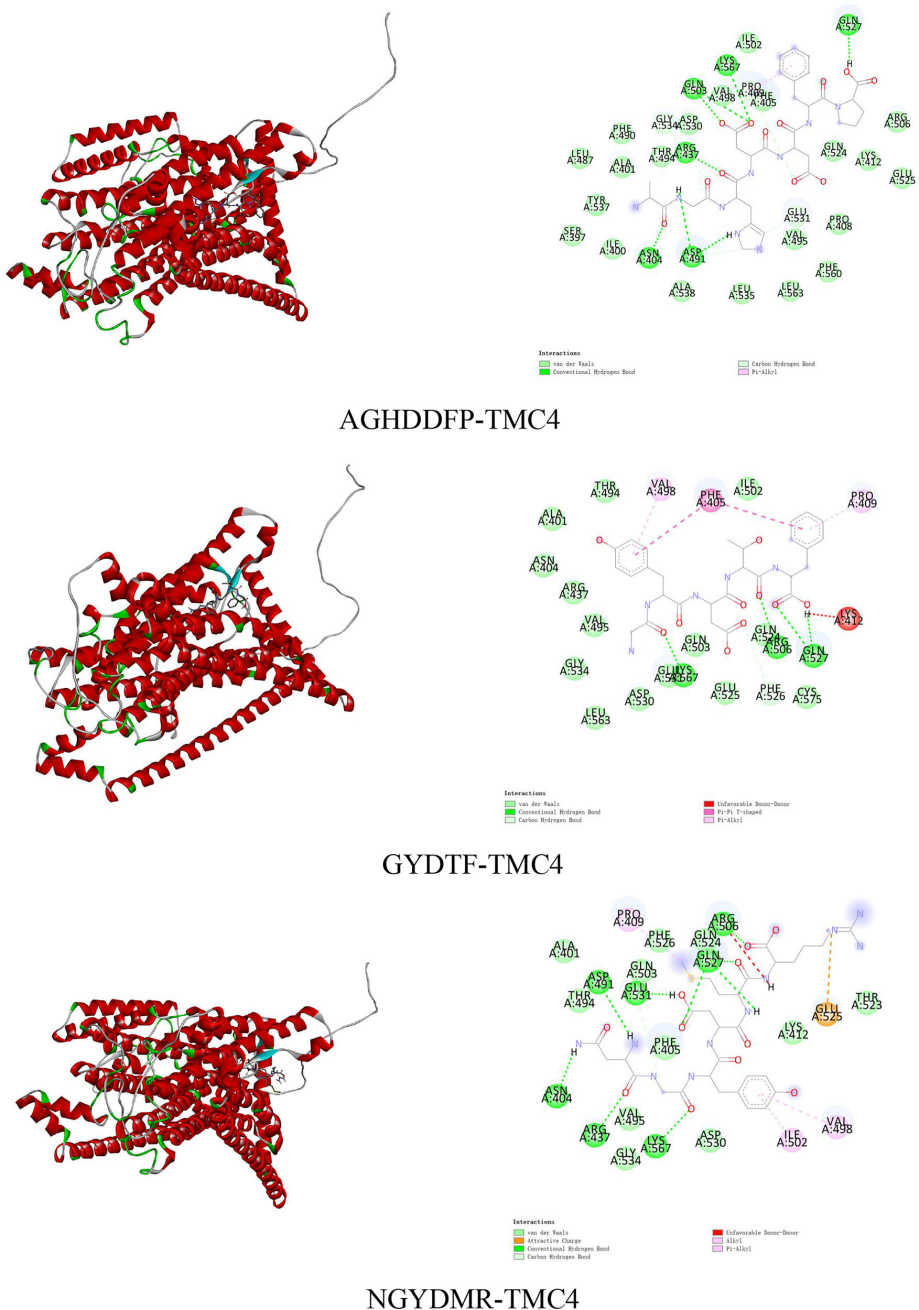
Interactions of AGHDDFP, GYDTF, and NGYDMR with TMC4.

Generally, a binding energy less than 0 indicated potential for spontaneous interaction, while values below −5.0 kcal/mol suggested a strong affinity between the ligand and target protein^1^. Details concerning the docking sites and energies for the 3 saltiness-enhancing peptides were presented in Table 2. As illustrated therein, the docking energies for the saltiness-enhancing peptides with TMC4 ranked as follows: AGHDDFP (−8.5 kcal/mol), GYDTF (−8.0 kcal/mol), and NGYDMR (−7.2 kcal/mol), demonstrating a strong affinity across all saltiness-enhancing peptides for TMC4. Table 2 also indicated that the active sites on TMC4 predominantly include residues Gln527, Glu531, Asp491, Asn404, Arg437, Lys567, Pro409, and Val498, with Gln527, Lys567, and Pro409 contributing significantly to the docking interactions observed with all saltiness-enhancing peptides. Furthermore, additional residues like Gln503, Phe405, Arg506, Phe526, Glu525, and Ile502 facilitated these interactions, albeit less frequently. Further analysis indicated that the predominant interactions between the saltiness-enhancing peptides and TMC4 included hydrogen bonding and hydrophobic interactions, supplemented by electrostatic forces (Fig. 4). These findings aligned with previous research underscoring the importance of these binding forces in ligand-receptor interactions^29^. Chen et al.^30^ found Gln527 as a key binding site in TMC4 for peptides derived from *L. edodes* while Wang et al. identified Gln527, Arg437, and Arg406 as critical in TMC4 for interaction with peptides from goose hemoglobin^31^. Our findings differed slightly from earlier studies which suggested residues such as Arg, Thr, and Tyr as pivotal in TMC4-peptide interactions^15,32^. This variance might be attributable to the varied amino acid compositions of the peptides and their respective docking pockets.

**Table 2:** Molecular docking energy and interacting amino acid residues between saltiness-enhancing peptides and TMC4

		GHDDFP	GYDTF	NGYDMR
Docking Energy with MC4(kcal/mol)		−8.1	−7.9	−7.9
Key Binding Sites of TMC4	Gln527	+	++	++
	Glu531	+		++
	Asp491	+++		+
	Asn404	+		+
	Arg437	++		+
	Gln503	++		
	Lys567	+	+	+
	Pro409	++	+	+
	Val498		+	+
	Phe405		++	
	Arg506		+	
	Phe526		+	
	Glu525			+
	Ile502			+

### Molecular dynamics simulations (MDS) analysis

**Fig. 5:**
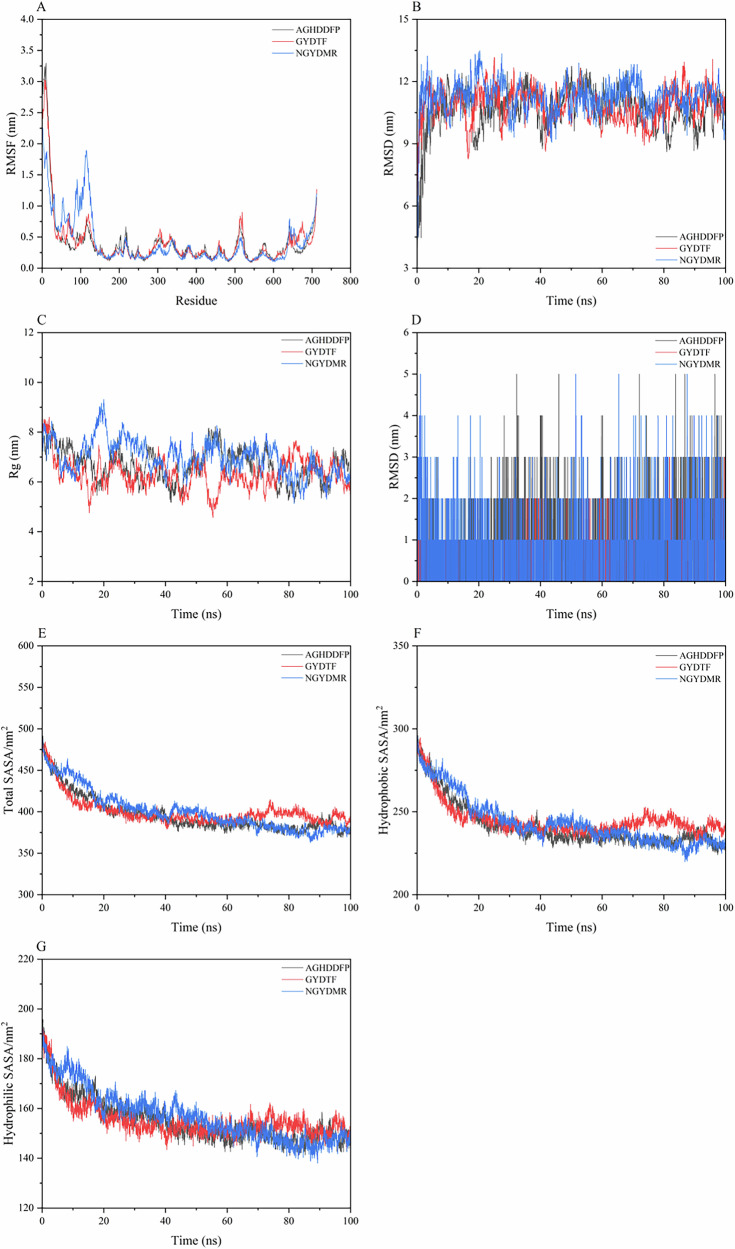
The results of molecular dynamics. **A** RMSF. **B** RMSD. **C** Rg. **D** The number of hydrogen bonds. **E** Total SASA. **F** Hydrophobic SASA. **G** Hydrophilic SASA.

**Fig. 6:**
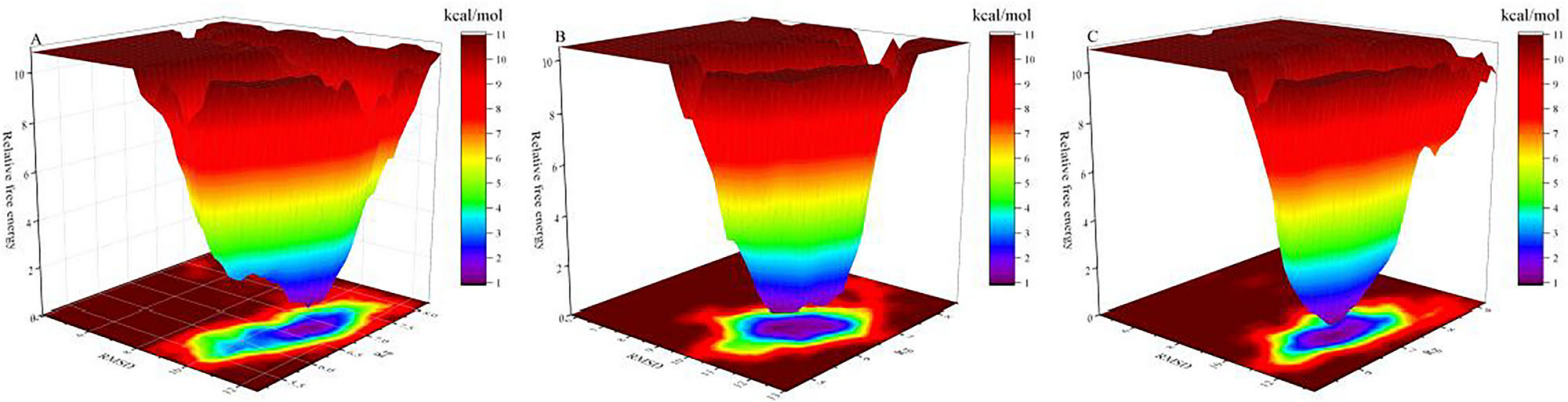
The free energy landscape map of 3 saltiness-enhancing peptides with TMC4. **A** The free energy landscape map of AGHDDFP-TMC4. **B** The free energy landscape map of GYDTF-TMC4. **C** The free energy landscape map of NGYDMR-TMC4.

To further explore the interactions between saltiness-enhancing peptides and TMC4, we conducted a 100 ns MDS of their complexes. The root mean square fluctuation (RMSF) provided insights into the amino acid residue dynamics within the complex, with higher RMSF values indicating greater conformational variability and flexibility^31^, the RMSF values fluctuated within a 0.6 nm range, suggesting relative stability (Fig. 5A). Residues around positions 200–300 in TMC4 exhibited significant fluctuations, suggesting enhanced flexibility in these regions. The root mean square deviation (RMSD) evaluated the overall system’s stability by measuring the positional deviations of all atoms from their initial states over time^33^. By 30 ns, the RMSD of the saltiness-enhancing peptide complexes bound to TMC4 had stabilized, with fluctuations remaining under 0.2 nm, indicating strong stability and tight binding (Fig. 5B). The compactness of the protein structure was assessed using the radius of gyration (Rg)^34^. which showed small fluctuations, less than 0.1 nm, confirming that the complexes maintained high compactness without significant conformational changes (Fig. 5C). Hydrogen bonding, a crucial interactive force, was analyzed to elucidate ligand influence on the complexes. Changes in hydrogen bond dynamics, shown in Fig. 5D, revealed relative stability, with the NGYDMR-TMC4 complex consistently forming 3–5 bonds. The counts for other complexes stabilized between 2 and 4 bonds, indicating that salt-enhancing peptides consistently formed at least two hydrogen bonds with active site residues, stabilizing the saltiness-enhancing peptides-receptor interaction^35^. Moreover, the solvent accessible surface area (SASA) was used to predict conformational changes upon ligand binding^36^. Figure 5E–G showed that the total SASA, hydrophobic SASA, and hydrophilic SASA of the complexes decreased, suggesting small conformational alterations and stable complex integrity. This stability likely results from extensive and effective hydrophobic interactions between TMC4 and the saltiness-enhancing peptides, promoting complex stability. Should the interaction between the receptor and ligand be relatively weak or unstable, the free-energy landscape typically showed multiple rugged minima. In contrast, strong and stable interactions were characterized by a single, smooth energy minimum in the potential energy profile. The free energy landscape for complexes of saltiness-enhancing peptides with TMC4 predominantly displayed a single and smooth minimum energy cluster, indicating strong stability (Fig. 6). In summary, the introduction of saltiness-enhancing peptides appeared to small impact the structural integrity of TMC4 while ensuring tight binding and enhanced stability.

In this study, a comprehensive analysis of 5984 peptides derived from the hydrolysate of *P. eryngii* (molecular weight <3 kDa) enabled the virtual identification of six candidate peptides with potential saltiness-enhancing properties. Subsequent sensory evaluations confirmed that each peptide exhibited different taste characteristics, with detection thresholds varying between 0.04 and 0.12 mmol/L. Notably, peptides AGHDDFP, GYDTF, and NGYDMR, when incorporated at a concentration of 1 mg/mL, significantly intensified the perception of saltiness in a 0.3% NaCl solution. Specifically, peptides AGHDDFP and GYDTF exhibited a synergistic interaction with NaCl, while NGYDMR showed an additive effect, which was consistent with electronic tongue. Molecular docking analysis demonstrated that these saltiness-enhancing peptides primarily engaged with TMC4 via hydrogen bonding, interacting with critical amino acid residues such as Gln527, Glu531, Asp491, Asn404, Arg437, Lys567, Pro409, and Val498. Subsequent MDS confirmed the formation of stable and tightly bound complexes between TMC4 and saltiness-enhancing peptides. Collectively, these findings offer a theoretical foundation for achieving sodium reduction in food systems and present novel insights into the potential application of *P. eryngii*.

## Methods

### Materials

*P. eryngii* was purchased was purchased from Fujian Zhongting Mushroom Industry Co., Ltd. Formic acid and acetonitrile were provided by Shanghai Macklin Biochemical Technology Co., Ltd. Six peptides (AGHDDFP, GYDTF, NGYDMR, FSDY, EMMETPPGF, and SPTP), each with a purity greater than 95%, were chemically synthesized by Shanghai Qiangyao Biotechnology Co., Ltd. (Shanghai, China).

### Preparation of *P. eryngii* hydrolysate (<3k Da) (PEU)

Initially, dried *P. eryngi*i was pulverized into a fine, uniform powder and passed through a 100-mesh sieve. The powder was then uniformly dispersed in ultrapure water at a ratio of 1:20 (w/v). The pH of the suspension was adjusted to 7.0 using food-grade citric acid and sodium bicarbonate. Enzymatic hydrolysis was subsequently conducted by adding flavourzyme (1000 U/g, final concentration 0.2%) and incubating the mixture at 55 °C for 4 h. After hydrolysis, the enzyme activity was terminated by boiling the reaction mixture for 15 min. The hydrolysate was then centrifuged at 8000 × *g* for 15 min at 4 °C to separate the clear supernatant. This supernatant was further purified through ultrafiltration using a membrane with a molecular weight cut-off of 3 kDa. Finally, the obtained filtrate (PEU) was lyophilized and preserved at −20 °C for further analysis.

### Peptide identification via Nano-LC-MS/MS

The PEU (1 mg/mL) was re-dissolved in solvent A (A: 0.1% formic acid in water) and analyzed by Orbitrap Fusion Lumos coupled to an EASY-nanoLC 1200 system (Thermo Fisher Scientific, MA, USA). 3 μL peptide sample was loaded onto a C18 column (20 cm × 75 μm i.d, 1.9 μm particle size) and separated with 60 min-gradient starting at 4% buffer B (80% acetonitrile with 0.1% formic acid) followed by a stepwise increase to 32% in 56 min, 90% in 0.5 min and stayed there for 3.5 min. The column flow rate was maintained at 300 nL/min with the column temperature of 40 °C. The electrospray voltage was set to 2 kV.

The mass spectrometer was run under data dependent acquisition mode, and automatically switched between MS and MS/MS mode. The survey of full scan MS spectra (m/z: 100–1500) was acquired in the Orbitrap with 120,000 resolution. The Normalized automatic gain control target of 200% and the maximum injection time of 100 ms. Then the precursor ions were selected into collision cell for fragmentation by higher-energy collision dissociation, the normalized collection energy was: 25%, 30%, 35%. The MS/MS resolution was set at 50,000, the Normalized automatic gain control target of 200%, the maximum injection time of 86 ms, and dynamic exclusion was 30 s.

### Virtual screening of potential saltiness-enhancing peptides

Umami compounds potentiated saltiness, and umami peptides have demonstrated saltiness-enhancing effects^19,37^. Accordingly, virtual screen of PEU peptides was conducted to identify umami candidates predicted to enhance perceived saltiness (referred to as potential saltiness-enhancing peptides). These candidates are henceforth referred to as potential saltiness-enhancing peptides^11^. Peptides achieving a PeptideRanker score above 0.5 were considered. Researches had demonstrated that shorter peptides, typically fewer than 10 amino acids, offered distinct taste characteristics. Other criteria for selection included the absence of toxicity and allergenicity, favorable water solubility, and good stability. The umami potential was evaluated utilizing multiple machine-learning models, such as Umami-SCM, UMPred-FRL, Umami-YYDS, and Umami-MRNN. Subsequently, these predictions were further validated by the BIO-UWM sensory modules designed specifically for peptides and amino acids^21^.

### Taste characteristics and thresholds

Taste characteristics of diverse peptides at a concentration of 1 mg/mL were evaluated using quantitative descriptive analysis (QDA). The sensory evaluation was conducted by a panel comprising 14 trained assessors, evenly distributed by gender and aged between 18 and 35 years. Participation was voluntary and informed, and assessors possessed considerable expertise in accurately quantifying and distinguishing intensities of various taste attributes, including sourness, sweetness, bitterness, saltiness, umami, and astringency^11^. The evaluation utilized a standardized sip-and-spit method, employing comparative analyses with established reference solutions detailed in Table S1. Additionally, the detection thresholds of peptides were identified using the triangle test methodology (the lowest concentration of peptides in an aqueous solution discernible from distilled water)^7^. According to the regulatory guidelines of Jianghan University’s Human Ethics Committee, formal ethical approval is not required for food sensory evaluation studies. All experimental procedures adhered strictly to the ethical standards specified in the Declaration of Helsinki. Furthermore, written informed consent was obtained from all assessors before participation in the sensory evaluation sessions.

### QDA

Solutions containing 3 mg/mL NaCl served as the control, while identical NaCl solutions supplemented with different peptides at a concentration of 1 mg/mL constituted the test samples. Reference solutions of NaCl at concentrations of 3, 7, and 11 mg/mL were designated saltiness intensity ratings of 5, 10, and 15, respectively. Aliquots of 10 mL of each sample were dispensed into transparent, odorless plastic bottles labeled with randomly assigned three-digit codes and presented to the assessors in a randomized sequence. Each assessor retained the solution in their mouth for 10 s during evaluation. To reduce residual taste effects and sensory fatigue, assessors rinsed their mouths twice with water between samples and took a 10 min rest interval. Saltiness-enhancing effects were identified based on QDA results. Peptides demonstrating significant enhancement of saltiness intensity were selected for further investigation.

### TI

The TI evaluation was performed in accordance with the protocol established in our prior research^12^. Prior to conducting the TI evaluation, assessors underwent an eight-day training regimen compliant with the ASTM E1909-97 Standard Guide for Time-Intensity Evaluation of Sensory Attributes. This preliminary training markedly enhanced the assessors’ proficiency in precisely quantifying saltiness intensity on a 0–15 scale, wherein 0 represented no perceivable saltiness and 15 corresponded to maximal saltiness perception. Saltiness reference standards employed were consistent with those detailed in Section 2.5.2. Additionally, assessors received comprehensive instruction on TI protocols and engaged in five product evaluation sessions, each lasting 2 h. During the evaluations, assessors utilized a graphical recording sheet featuring time (seconds) on the x-axis and saltiness intensity (0–15) on the y-axis. Intensity measurements were recorded at specific time intervals of 5, 10, 20, 30, 45, 60, 75, 90, and 105 s. Qualification as an assessor required achieving at least 40% consistency in TI curves, following the criteria set forth by Goodstein et al.^38^.

The control consisted of a 3 mg/mL NaCl solution, while the experimental samples comprised a combination of the NaCl solution (3 mg/mL) with one of three peptides (AGHDDFP, GYDTF, and NGYDMR) at a concentration of 1 mg/mL. Each assessor was instructed to ingest 10 mL of the sample, retain it orally for 10 s, and subsequently expectorate. Saltiness intensities were documented at predetermined intervals until saltiness was no longer perceptible. A mandatory 20-min resting interval was enforced between each evaluation session, during which assessors thoroughly rinsed their oral cavities with purified water. Sample presentation order was randomized, and each experiment was replicated three times to ensure the reliability and reproducibility of results.

### Interactions of saltiness-enhancing peptides with NaCl

The saltiness-enhancing peptides that enhanced saltiness were subjected to an S-curve analysis to elucidate their interaction types with NaCl, incorporating modifications from previous methodologies^19^. We devised a series of mixed aqueous solutions that blend 3 saltiness-enhancing peptides with NaCl at varied concentrations. Each sample underwent sensory evaluation employing a three-alternative forced-choice (3-AFC) methodology. A sigmoidal model was utilized to characterize the correlation between stimulus concentration and detection probability (P), enabling the generation of concentration-detection probability (C-P) curves. The experimental threshold, identified as the concentration at which detection probability reached 0.5, was then compared to the theoretical threshold. This comparison elucidated the nature of interactions occurring between saltiness-enhancing peptides and NaCl.

### Electronic tongue

Electronic-tongue analyses were conducted on an Insent TS-5000Z equipped with six taste sensors, umami (AAE), astringency (AE1), bitterness (C00), sourness (CA0), saltiness (CT0), and sweetness (GL1). Before testing, the instrument was calibrated with distilled water and 0.01 mol/L HCl; 0.01 mol/L NaCl and 0.01 mol/L monosodium glutamate served as standard solutions. Throughout testing, samples were maintained at a constant temperature of 25 °C. The control solution was 3 mg/mL NaCl. Test solutions were prepared by supplementing the NaCl solution with individual peptides at 1 mg/mL each (AGHDDFP, GYDTF, NGYDMR, FSDY, EMMETPPGF, and SPTP). Each sample was measured four times, excluding the initial measurement due to its high variability. Measurement session was 30 s. Saltiness response value was quantified based on the differential electrical potentials generated by each sample.

### Homology modeling and molecular docking

Homology modeling and molecular docking were conducted following the established method described previously^11^. The three-dimensional (3D) structure of TMC4 was generated by homology modeling using TMC1 (PDB ID: 7USW) as the structural template. The reliability and accuracy of the constructed TMC4 model were evaluated by utilizing PROCHECK. Following this, molecular docking of peptides to TMC4 was conducted utilizing Autodock Vina software. This was succeeded by evaluations of binding energies, interaction sites, and conformational modes.

### Molecular dynamics simulations

In this study, ligand topology files were generated utilizing the sobtop-1.0 (dev3.1) platform, while protein structures were subsequently refined through the SPDBV-4.10 software. Charge computations were conducted employing orca-5-0-4 alongside Multiwfn-3.8-dev.23^39^. Receptor-ligand complexes, involving TMC4 and saltiness-enhancing peptides produced via molecular docking, underwent further examination through MDS executed in Gromacs2024. The proteins and ligands were parameterized using amber99sb.ff and Gaff force fields, respectively. MDSs spanned 100 ns under standardized conditions of temperature and pressure.

### Statistical analysis

The results obtained from triplicate experiments are expressed as mean ± standard deviation. Statistical analyses were performed utilizing SPSS software (Version 26.0, IBM Corp., Armonk, NY, USA). Variations among experimental groups were evaluated using analysis of variance (ANOVA), followed by Duncan’s multiple range test to determine statistically significant differences.

## Supplementary information

Supporting information

## Data Availability

This manuscript does not report data generation or analysis.
