# Supplementary material for: Characterization of saltiness-enhancing peptides from Pleurotus eryngii: identification, sensory evaluations, and mechanism of saltiness-enhancing
Source: NPJ Sci Food. 2026 Feb 7;10:39. doi: 10.1038/s41538-025-00681-8 (PMC12887059; doi:10.1038/s41538-025-00681-8)
Supplement: Supplementary file 1 — Supporting information [file 41538_2025_681_MOESM1_ESM.docx]

**Supporting information**

**Characterization of saltiness-enhancing peptides from *Pleurotus eryngii*: Identification, sensory evaluations, and mechanism of saltiness-enhancing**

Min Yang ^a,1^, Wei Wang ^a,1^, Biyang Zhu ^b^, Changli Zeng ^a^, Aimin Ma ^c^, Hongbo Wang ^a*^, Danyun Xu^a*^

^a^ College of Life Science, Jianghan University, Wuhan 430056, Hubei, China

^b^ College of Light Industry and Food Engineering, Guangxi University, Nanning 530004, China

^c^ College of Food Science and Technology, Huazhong Agricultural University, Wuhan 430056, China

*Corresponding author: Danyun Xu

E-mail: xdy2075@jhun.edu.cn

^1^ These authors contributed equally.

**
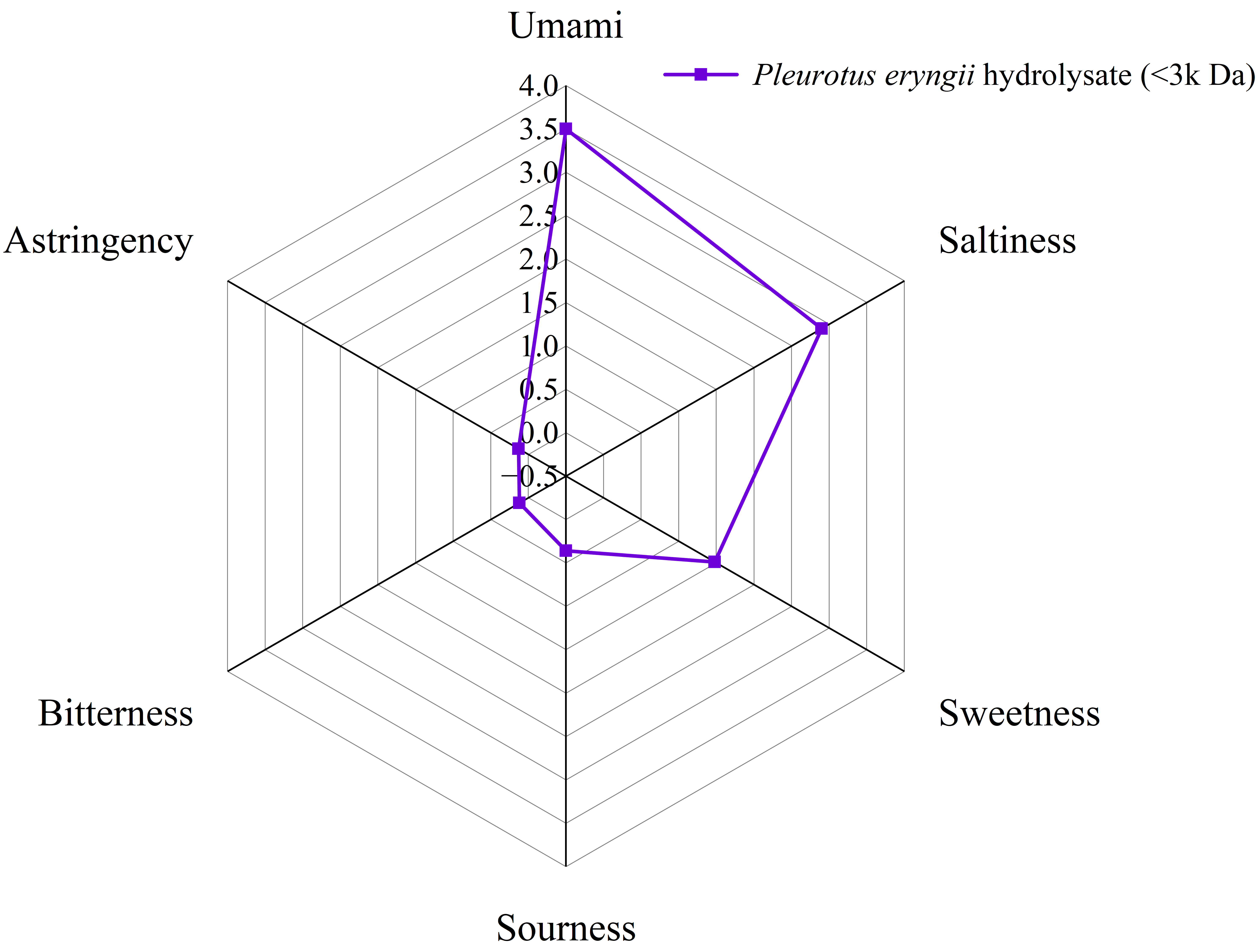
 Figure S1** Sensory evaluation of *Pleurotus eryngii* hydrolysate (<3k Da).


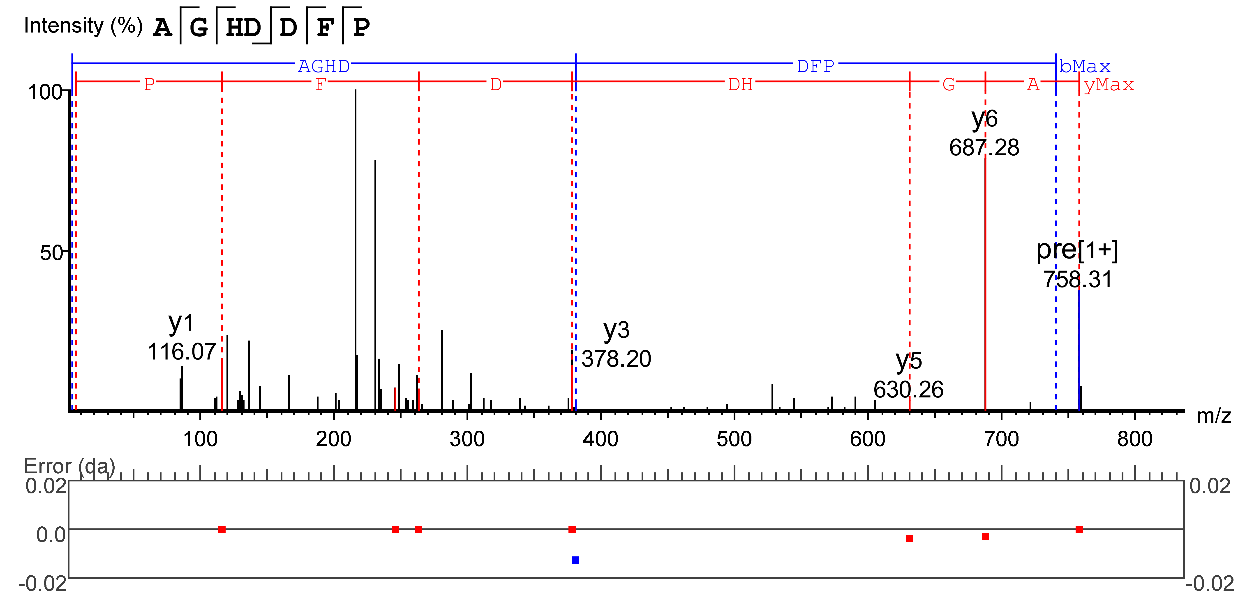


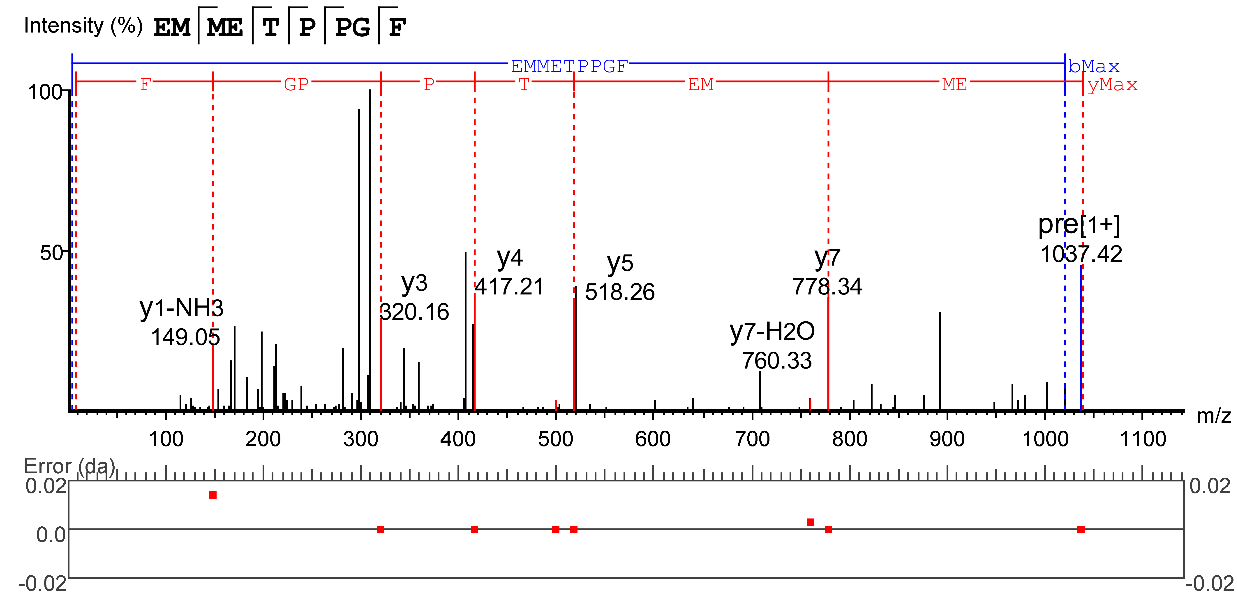


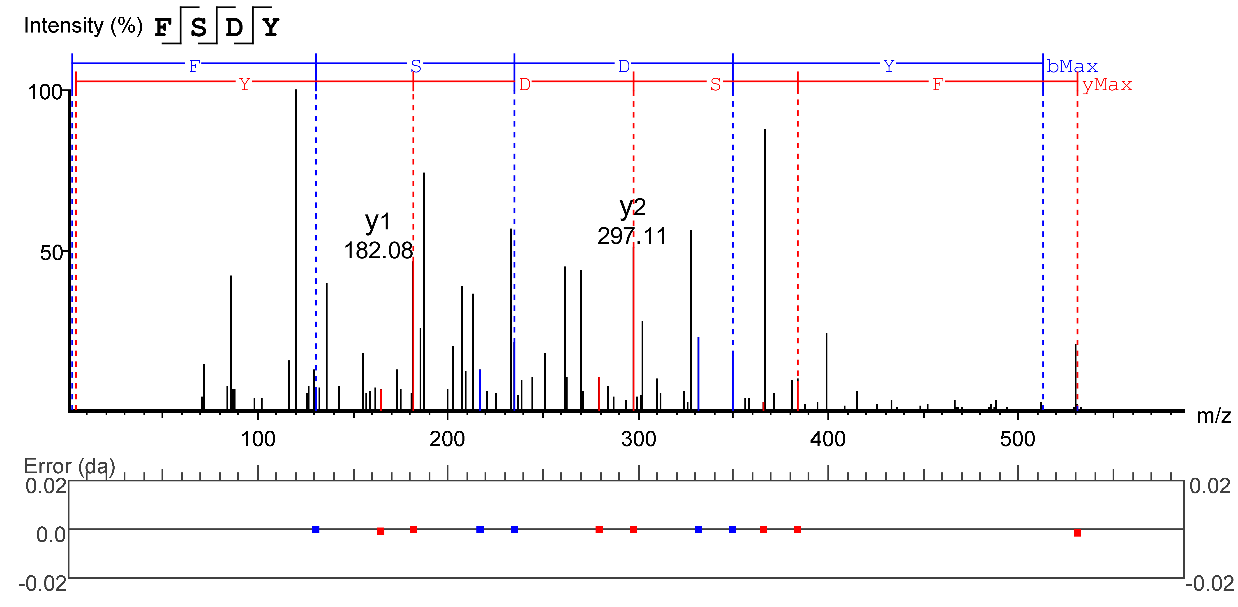


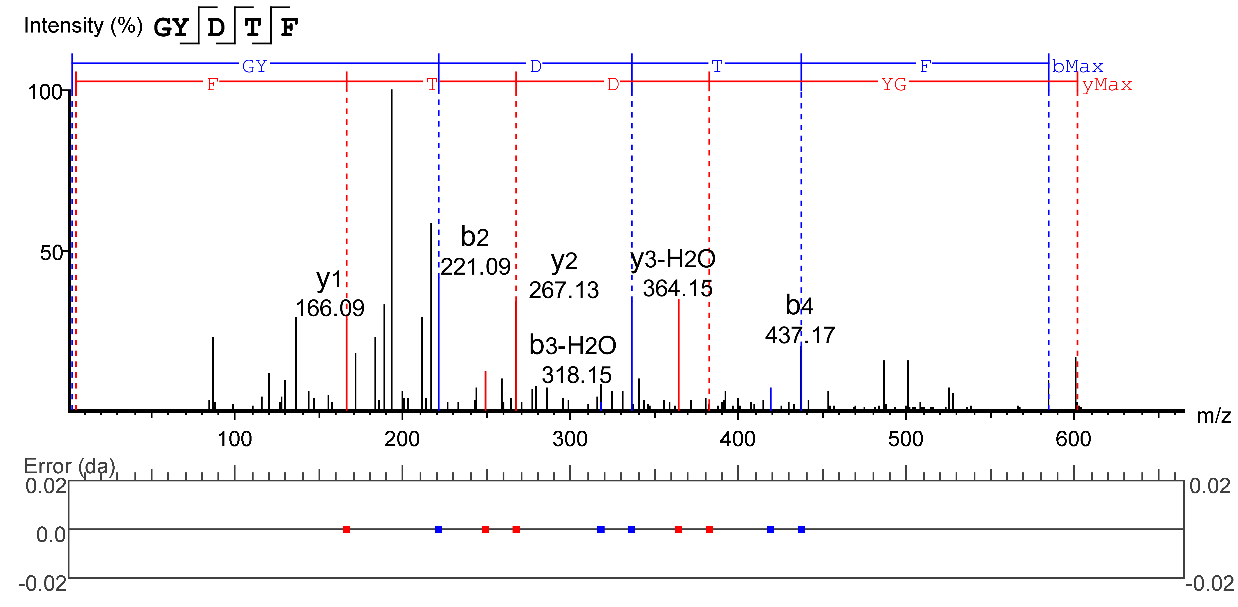


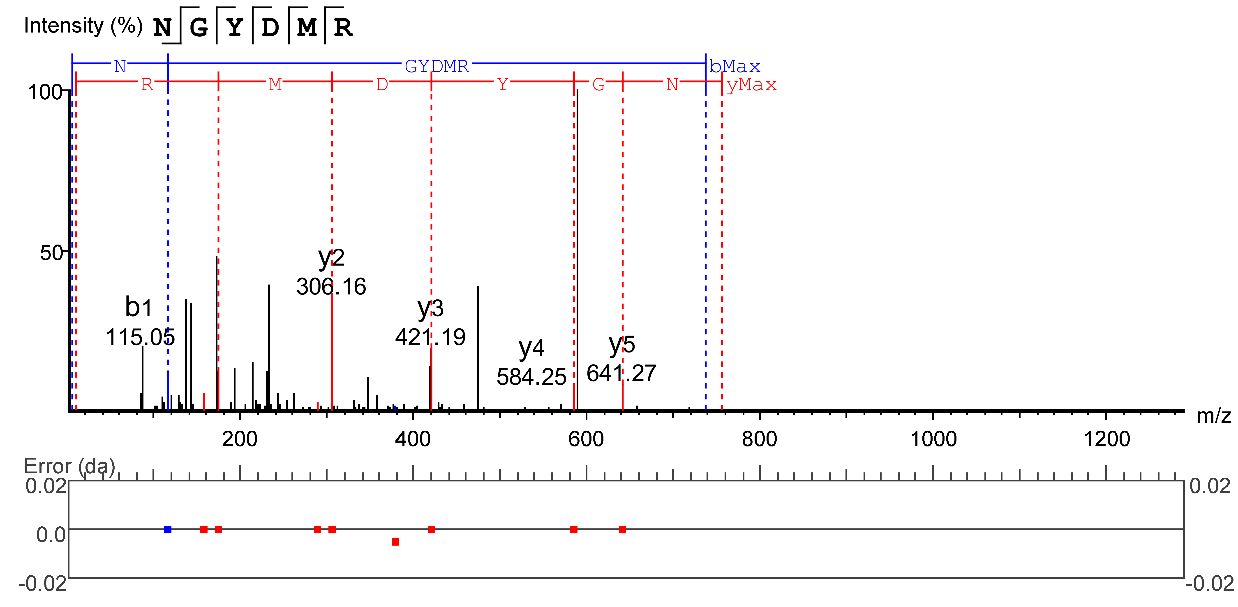


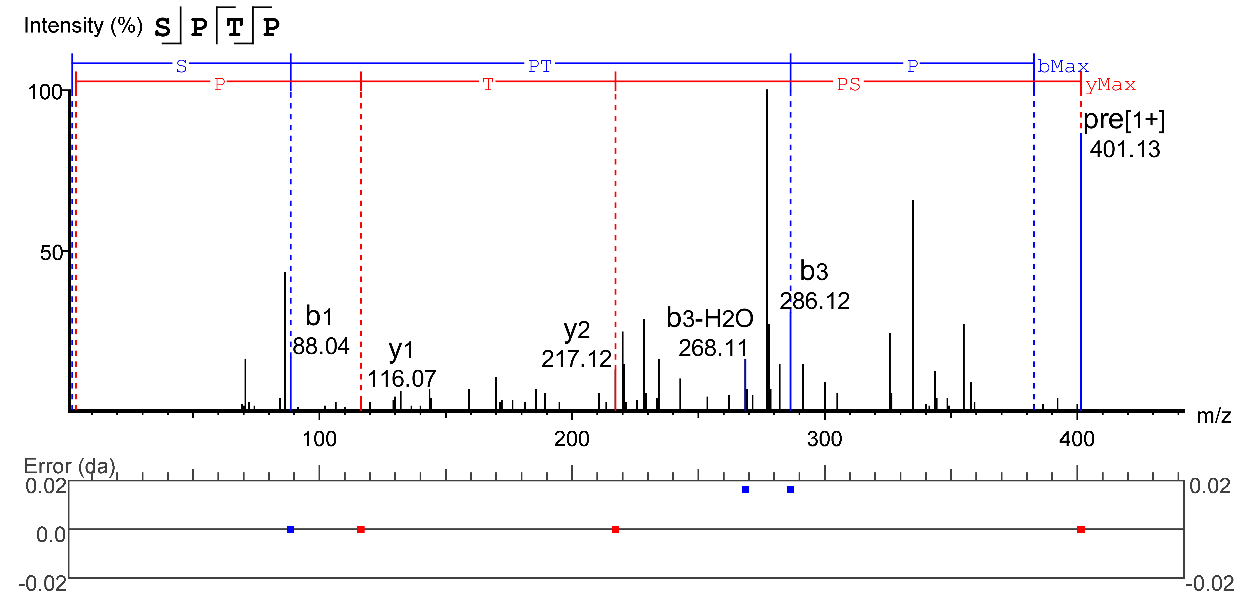


**Figure S2** MS/MS spectrum of the identified 6 peptides (AGHDDFP, GYDTF, NGYDMR, FSDY, EMMETPPGF, and SPTP).

=


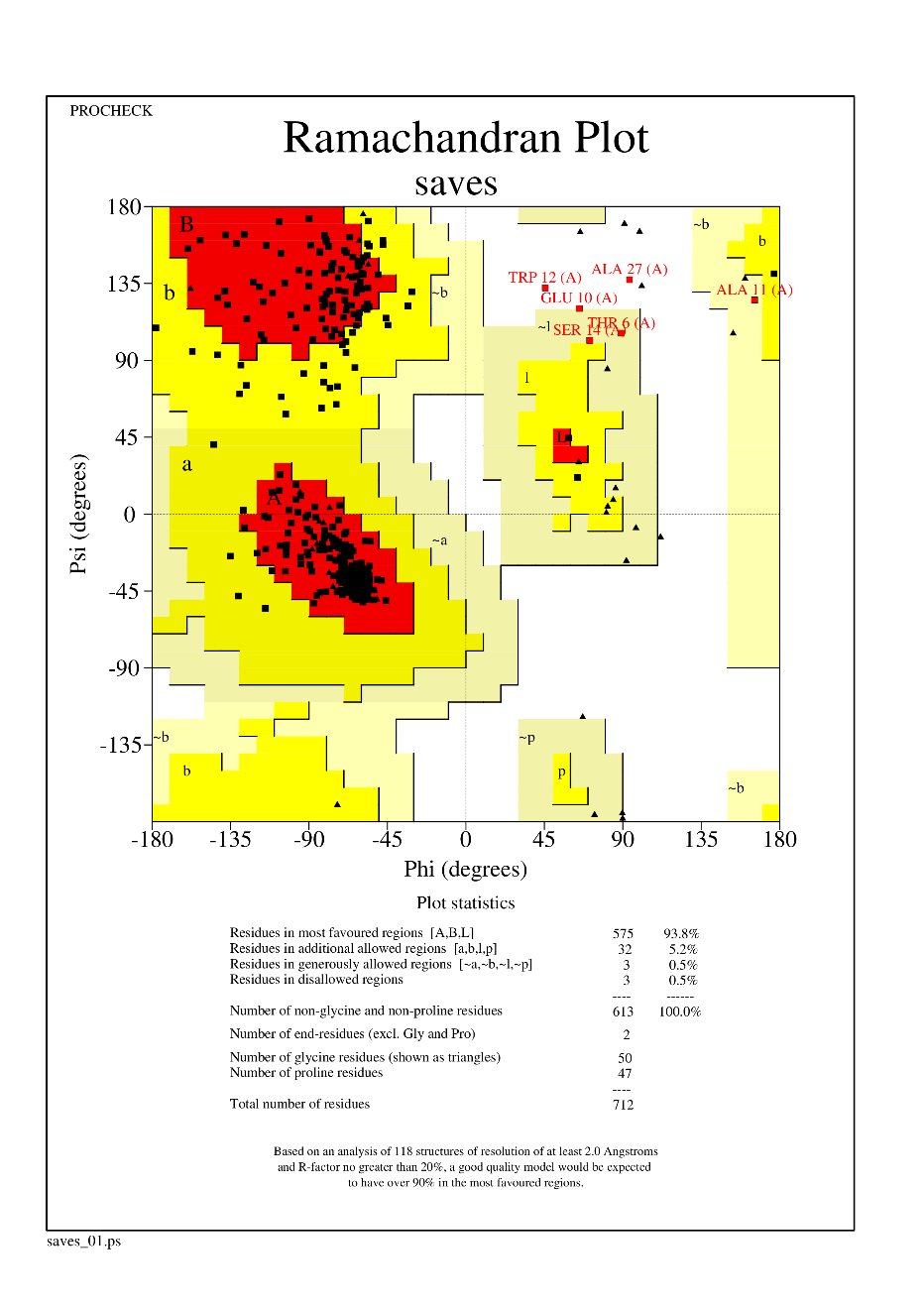


**Figure S3** Ramachandran plot of homology model for TMC4.

**Table S1** Sensory evaluation criteria

| Taste | Standard compound | Score and corresponding concentration (mg/mL) | | |
| --- | --- | --- | --- | --- |
|  |  | 5 | 10 | 15 |
| Umami | MSG | 3 | 5 | 7 |
| Saltiness | NaCl | 3 | 5 | 7 |
| Sourness | Citric acid | 0.25 | 0.5 | 0.75 |
| Sweetness | Sucrose | 2 | 4 | 6 |
| Bitterness | L-isoleucine | 2.5 | 5 | 7.5 |
| Astringency | Tea polyphenols | 0.2 | 0.4 | 0.6 |

**Table S2** Means of the sensory panel for the parameters extracted from TI curves for umami

| Parameter | CK | AGHDDFP | GYDTF | NGYDMR |
| --- | --- | --- | --- | --- |
| I _max_ | 5.50±0.32^a^ | 8.83±0.41^c^ | 8.58±0.38^c^ | 7.25±0.27^b^ |
| T _start_ | 5±0^a^ | 5±0^a^ | 5±0^a^ | 5±0^a^ |
| T _max_ | 30±0^b^ | 20±0^a^ | 20±0^a^ | 20±0^a^ |
| T _plateau_ | 15±0^b^ | 10±0^a^ | 10±0^a^ | 10±0^a^ |
| T _ext_ | 75±0^a^ | 102.50±6.12^c^ | 90±0^b^ | 90±0^b^ |
| R _increase_ (×10^-2^) | 17.30±2.07^a^ | 37.20±1.37^bc^ | 38.90±1.72^c^ | 36.10±1.36^b^ |
| R _decrease_ (×10^-2^) | -10.60±0.86^b^ | -10.10±1.22^b^ | -10.80±0.53^b^ | -8.10±0.43^a^ |
| Area _before_ (×10^2^) | 2.82±0.12^d^ | 2.50±0.13^c^ | 2.32±0.12^b^ | 1.92±0.09^a^ |
| Area _after_ (×10^2^) | 2.23±0.24^a^ | 9.42±0.77^d^ | 6.60±0.57^c^ | 5.06±0.33^b^ |
| Area (×10^2^) | 5.04±0.33^a^ | 11.95±0.70^d^ | 8.92±0.66^c^ | 6.98±0.34^b^ |

CK, control group. Results are presented as mean ± standard deviation. Columns with different letters indicate a significant difference (P < 0.05) according to Duncan’s multiple range test.

**Table S3** Definitions of 10 parameters extracted from TI curve.

| Attribute | Definition |
| --- | --- |
| 1 | Maximum perceived intensity |
| T _start_ | The time when the attribute was first perceived |
| T _max_ | The time elapsed to maximum intensity |
| T _plateau_ | Time duration which the intensity is ≥ 90% of Imax |
| T _ext_ | Extinction time, when the sensation returns to zero |
| R _increase_ | Rate of intensity increase before Imax |
| R _decrease_ | Rate of intensity decrease after Imax |
| Area _before_ | Area under the curve before maximum |
| Area _after_ | Area under the curve after maximum |
| Area | Area under the overall curve |
